# Highly efficient generation of biallelic reporter gene knock-in mice via CRISPR-mediated genome editing of ESCs

**DOI:** 10.1007/s13238-015-0228-3

**Published:** 2015-12-10

**Authors:** Yanliang Wang, Junhong Li, Jinzhu Xiang, Bingqiang Wen, Haiyuan Mu, Wei Zhang, Jianyong Han

**Affiliations:** State Key Laboratories for Agrobiotechnology, College of Biological Sciences, China Agricultural University, Beijing, 100083 China

**Dear Editor,**

Targeted gene knock-out and knock-in mice are valuable tools for elucidating the function of genes *in vivo* (Capecchi, 2001). Recently, the Cas9 endonuclease from *Streptococcus pyogenes* type II CRISPR system has been demonstrated as a powerful tool for gene targeting. Under the guidance of a synthetic 20-nucleotide single guide RNA (sgRNA), Cas9 protein can bind to specific genome locus and generate targeted double-stranded break (DSBs) to facilitate efficient genome editing (Cong et al., 2013; Mali et al., 2013). A one-step method has been reported to generate gene-targeted knock-in mice by injecting Cas9 mRNA in combination with a sgRNA and single-stranded DNA oligo complex/construct into the cytoplasm of a zygote (Hai et al., 2014; Wang et al., 2013; Yang et al., 2013). However, it is inefficient to generate knock-in mice that carry a reporter gene. Using Cas9 protein combined with mRNA of dual-crRNA and tracrRNA has greatly increased the efficiency (Aida et al., 2015), but in most mice resulted in the modification of a single allele. There were few reports on the generation of reporter knock-in mice from embryonic stem cells (ESCs) that were genetically modified by the CRISPR/Cas9 system. Here, we report an effective method, combining the CRISPR/Cas9 system and eight cell-stage embryo injection technology, leading to rapid generation of biallelically modified reporter knock-in mice within one month.

We chose *Tbx3* as the candidate gene to demonstrate the feasibility of this method because we have reported that Tbx3 can improve the quality of induced pluripotent stem cells. To protect the integrity of Tbx3 function, we designed sgRNA targeting site just upstream the stop codon of *Tbx3* and used the self-cleaving 2A peptide for mediation of the fusion between Tbx3 and GFP (*Tbx3*-2A-GFP) (Fig. 1A). A plasmid containing Cas9 and sgRNA was transfected into ES cells (G4 cell line), and T7 endonuclease I (T7EI) assay verified a target site efficiency of 46% (Fig. 1B). For generating *Tbx3*-2A-GFP ESCs, we transfected ESCs with the CRISPR expression vector combined with the *Tbx3*-2A-GFP donor plasmid. Tbx3 expression pattern in ESCs is heterozygous and most cells are Tbx3-negative. Addition of two small-molecule inhibitors (2i) of mitogen-activated protein kinase (MAPK) and glycogen synthase kinase 3 (GSK3) pathways facilitated a homogenous Tbx3 positive ESC stage (Fig. S1A), which were helpful for sorting correct knock-in ESCs. Therefore, we supplemented the media with 2i the day following transfection. GFP expression was detected as early as 48 h post-transfection (Fig. 1C). Flow cytometry analysis indicated that 7.9% transfected cells were GFP-positive (Fig. S1B). Introduction of resistance gene into CRISPR expression plasmid and drug selection after transfection increased the efficiency up to 17.2% (Fig. S1B). Each single GFP-positive cell was sorted into one well of a 96-well plate by fluorescence-activated cell sorting (FACS) technology. We generated a total of 24 clones after FACS, 2 of which were GFP-negative. A PCR assay was performed, on 11 out of the other 22 GFP-positive clones, and the results verified that 10 out of the 11 clones had modification on both alleles (Fig. S1D). These results demonstrate that biallelically modified ESCs can be efficiently generated by combinatorial use of both CRISPR/Cas9 system and FACS technology.

**Figure 1:**
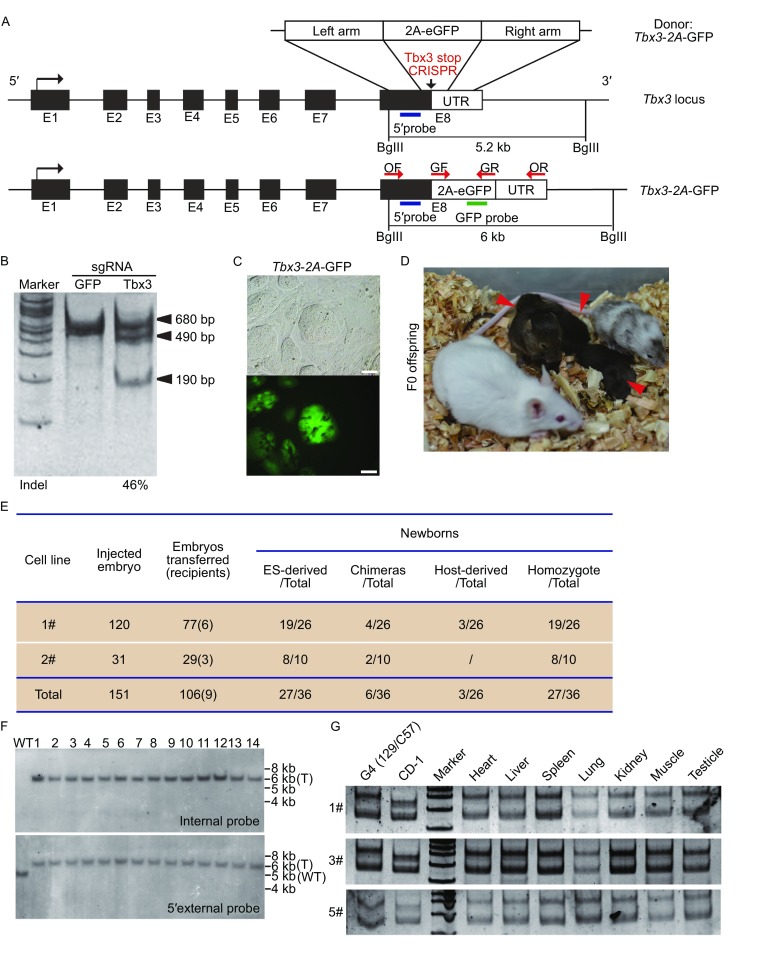
**Eight-cell stage embryo injection technology facilitates the generation of biallelic GFP cassette knock-in mice**. (A) Schematics depicting the targeting strategy for generation of *Tbx3-2A-GFP* knock-in ESCs. UTR, untranslated region of *Tbx3*. (B) T7 endonuclease I (T7EI) assay for CRISPR/Cas9 medaite cleavage at the Tbx3 locus in ESCs. The result is shown in the lower panel. (C) Up and bottom pannel show the phase contrast image and corresponding GFP fluorescence image of *Tbx3*-2A-GFP ESCs. Scale bars, 50 µm. (D) F0 generation of *Tbx3-2A-GFP* mice generated by eight-cell stage embryo injection (marked by red arrowheads). (E) Summary of generation of *Tbx3*-2A-GFP mice (F) Identification of F0 generation knock-in mice by Southern blot analysis. WT, wild type; T, targeted mice. (G) Microsatellite analysis of tissues from *Tbx3-2A-GFP* mice using D1Mit132 primers

To test whether the reporter knock-in ESCs retain the pluripotent potential after genetic manipulation, we first performed immunofluorescence staining. As shown in Fig. S1C, *Tbx3-2A-GFP* ESCs expressed key pluripotency markers such as Oct4, Nanog and Sox2. ESCs have the capacity to generate all cell types of an adult organism. These three germ lineages could be effectively induced after lineage differentiation by standard EB formation, following immunofluorescence staining for the differentiated cells on day 7 (Fig. S1E). These results indicate that reporter knock-in ESCs retain the pluripotent potential and the capacity to form three germ lineages following genetic manipulation by the CRISPR/Cas9 system.

Compared with conventional injections of ESCs into blastocyst host, eight cell-stage embryo injection efficiently yields F0 generation mice, allowing for immediate phenotypic analyses (Poueymirou et al., 2007). To generate *Tbx3-2A-GFP* mice, the reporter knock-in ESCs were injected into the perivitelline space of the eight cell-stage embryos with the help of Piezo. A total of 151 embryos were injected with two ES cell lines with biallelic modification in three independent experiments. Of the 151 embryos, 106 (70.2%) embryos developed into blastocyst or morula stages (Fig. 1E), with the majority of blastocyst embryos containing an ES-derived ICM (Fig. S2A). All injected embryos were transferred into seven pseudopregnant CD1 females in three independent experiments. Six recipient females were pregnant and delivered a total of 36 newborns. Of the 36 newborn F0 generation, 27 were male with 100% ESC derived coat color, 3 were male or female with 100% host-derived coat color, and 6 were chimeras. Southern blot analysis showed that all males in 100% ESC-derived coat color were carrying the GFP transgene in both *Tbx3* alleles, which is further confirmed by microsatellite assay of tissues using D1Mit132 primers that reporter knock-in mice were from ESCs (Fig. 1F and 1G). We next determined the transmission rate of *Tbx3*-2A-GFP mice by crossing the F0 generation males with wild-type CD1 females. All newborns manifested 100% ESC-derived coat color (Fig. S2C), and early stage embryos (E10.5 and E13.5) have a consistent expression pattern of GFP (Fig. S2D and S2E). Taken together, our results support the conclusion that eight cell-stage embryo injection can yield nearly 100% ESC-derived newborns (Poueymirou et al., 2007), and demonstrates that the combination of the CRISPR/Cas9 system with eight-cell stage embryo injection technology can quickly and efficiently generate biallelic modified reporter knock-in mice.

We further examined whether the reporter knock-in mice can be used for phenotypic analysis immediately. Fluorescence observation of reporter knock-in mice at embryonic day (E) 13.5 after embryo transfer revealed that GFP can be detected in the skeleton, eye, lungs, mammary gland, limb, hair follicle, mandibular and maxillary region, which is consistent with a previous study (Fig. 2D) (Chapman et al., 1996). However, during development of the lungs, expression of Tbx3 in the lung mesenchyme has been found to require additional regulating elements (Horsthuis et al., 2009). Consistent with this report, immunohistochemistry analysis of serial sections of the lungs confirmed that GFP is expressed in the mesenchyme (Fig. 2E).

**Figure 2:**
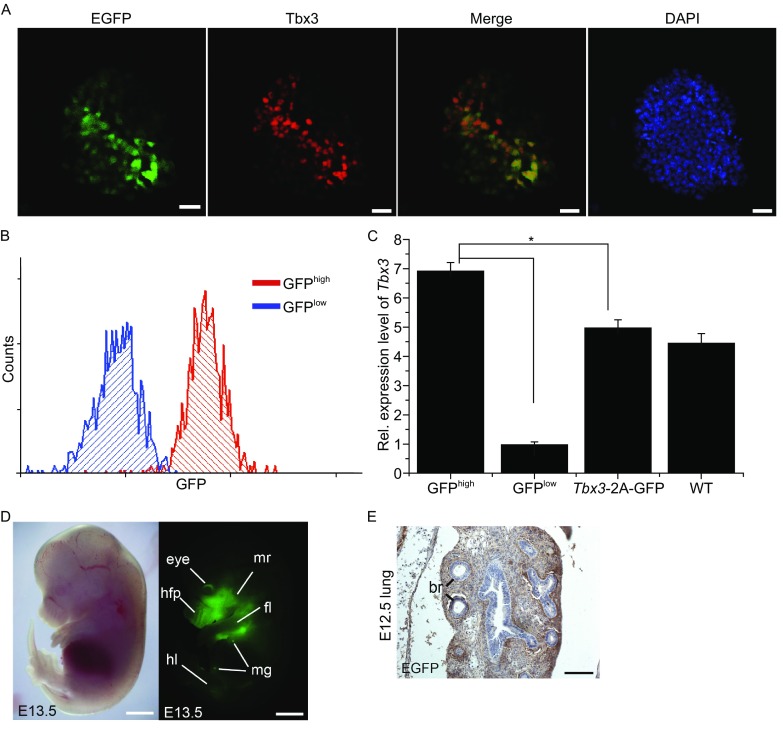
**Both** ***Tbx3-2A-GFP*** **mice and ESCs can be used for phenotypic analysis directly**. (A) The consistent expression pattern of GFP and Tbx3 in *Tbx3-2A-GFP* ESCs. Scale bars, 50 µm. (B) *Tbx3-2A-GFP* ESCs were sorted into two subpopulations based on the fluorescence intensity of GFP. (C) The expression levels of *Tbx3* in two subpopulations were quantified by RT-PCR. (D) Phase contrast images (left) and corresponding GFP fluorescence images (right) of early embryos and tissues from *Tbx3*-2A-GFP mice. fl, forelimb; hl, hindlimb; ml, milk line; mr, mandibular and maxillary region; mg, mammary gland; hfp, hair follicle placodes. Scar bars: 1 mm. (E) Immunohistochemical detection of GFP in the section of lung from knock-in mice. br, bronchi. Scar bars, 50 µm

Heterogeneous gene expression is one of the unique properties of ESCs (Niwa et al., 2009). High-quality knock-in ESCs carrying a reporter gene cassette is a powerful tool for elucidating the molecular basis of early embryo development. To test whether *Tbx3*-2A-GFP ESCs can be used for such study, we first examined the expression patterns of Tbx3 and GFP in the culture medium without 2i. Immunofluorescence staining results confirmed that GFP had a consistent expression pattern with Tbx3 (Fig. 2A). Next, *Tbx3*-2A-GFP ESCs were sorted into two subpopulations according to their fluorescence intensity. Quantitative PCR (q-PCR) analysis showed that the GFP^high^ population had higher expression of Tbx3 than GFP^low^ population and the insertion of *GFP* had no effect on the expression of *Tbx3* (Fig. 2B and 2C). Taken together, our results demonstrate that Tbx3-2A-GFP mice and ESCs can be used for direct phenotypes analysis.

Recent studies in human cell lines demonstrated that CRISPR/Cas9 system has a potential off-target cleavage in a sequence and position-dependent manner, since Cas9 protein can tolerate small numbers of mismatches between sgRNA and target DNA (Fu et al., 2013; Hsu et al., 2013). To test whether there were potential off-target cleavages in *Tbx3-2A-GFP* mice, we predicted 11 potential off-target sites throughout the genome containing up to three mismatches when compared with the 20 bp sgRNA coding sequence (Table S1). We randomly selected ten mice for off-target analysis, and genome regions flanking potential off-target sites was amplified and tested for potential off target mutation by T7EI assay. No mutation was detected at any locus of these mice, but we could not exclude the existence of off-target cleavages due to the sensitivity limitation of T7EI assay. However, our results are consistent with the report that up to three mismatches can eliminate the detectable Cas9 cleavage (Hsu et al., 2013).

In summary, we have shown that the CRISPR/Cas9 system can efficiently execute site-specific insertion of a reporter cassette in ESCs, and then quickly generate biallelic modified mice by injecting boallelic modified ESCs into eight-cell stage embryo (Fig. S3). Using this method, we can rapidly generate reporter knock-in mice and ESCs at the same time, which allows the investigation of the molecular basis of early embryo development both *in vivo* and *in vitro* to be performed simultaneously. This approach has several advantages. First, FACS sorting can be readily used to identify correctly targeted cells. When sorting GFP-positive cells, we chose to select those with higher fluorescence intensity. Our selective screening process was based on the assessment that biallelic GFP cassette knock-in would indicate a higher fluorescence intensity, when compared with monoallelic insertion. Second, ESCs were cultured in the presence of two small-molecule inhibitors (2i). The quality of ESCs is an essential determinant for eight-cell stage embryo injection. The supplementation with 2i can maintain mESCs in the ground state, bearing resemblance to preimplantation mouse epiblasts (Ying et al., 2008). However, this method has its limitations. The method we reported here can effectively generate biallelic modified reporter knock-in mice, but this method is only suitable for genes expressed in ESCs, and further improvement will be needed.

## Electronic supplementary material

Below is the link to the electronic supplementary material.

Supplementary material 1 (PDF 440 kb)
