# Erratum to: Highly efficient generation of biallelic reporter gene knock-in mice via CRISPR-mediated genome editing of ESCs

**DOI:** 10.1007/s13238-017-0392-8

**Published:** 2017-03-10

**Authors:** Yanliang Wang, Junhong Li, Jinzhu Xiang, Bingqiang Wen, Haiyuan Mu, Wei Zhang, Jianyong Han

**Affiliations:** State Key Laboratories for Agrobiotechnology, College of Biological Sciences, China Agricultural University, Beijing, 100083 China

## Erratum to: Protein Cell (2016) 7(2):152–156 DOI 10.1007/s13238-015-0228-3

In the original publication the supplementary figures have been missed out in the supplementary file section. The correct supplementary figures are provided in the link below.

## Electronic supplementary material

Below is the link to the electronic supplementary material.

Supplementary material 1 (DOCX 902 kb)

