# Supplementary material for: Highly efficient generation of biallelic reporter gene knock-in mice via CRISPR-mediated genome editing of ESCs
Source: Protein Cell. 2015 Dec 10;7(2):152–6. doi: 10.1007/s13238-015-0228-3 (PMC4742388; doi:10.1007/s13238-015-0228-3)
Supplement: Supplementary file 1 — Supplementary material 1 (PDF 440 kb) [file 13238_2015_228_MOESM1_ESM.pdf]

This supplementary data file contains the following contents: Materials and methods, Supplementary Figures (S1~S3) and Supplementary Tables (S1~S5).

Materials and methods

Animal experiments

All animal works were proceeded according to the Guide for the Care and Use of Laboratory Animals. We used Vr:CD1 (ICR) mice as the embryo donor and recipient, which were purchased from Vital River Laboratories Company (Beijing, China). All mice were housed in specific pathogen free (SPF) grade cleanroom and maintained on a 12 hour dark/ 12 hour light cycle.

CRISPR/Cas9 plasmid

CRISPR expression plasmid targeting Tbx3 locus was constructed as previously published study (Cong et al., 2013), the px330 plasmid was a gift from Dr. Zhang Feng (Broad Institute of MIT and Harvard), oligonucleotide for sgRNA were synthesized by Sangon Biotech Company (Shanghai, China).

The method for construction of CRISPR expression plasmid containing PGK-puro cassette, called px330-puro, was described as follow: first, using XhoI and XbaI to subclone the PGK-puro cassette into the SalI and XbaI sites of pCS2-TALEN-A-perr plasmid (a gift from Dr. Zhang Bo, Peking University) forming intermediate plasmid, which

called pCS2-puro. Second, digest and subclone sgRNA/Cas9 cassette of px330 into pCS2-puro by AflIII and NotI.

Targeting vector

To construct donor vector, we amplified chromosome sequence flanking the stop codon of Tbx3 locus as homology arms. When designing primers for amplifying 5'-homologous arm, we introduced degenerate bases to remove CRISPR targeting site within the donor vector. Through fusion PCR, eGFP was fused with 5'-homologous arm preceded by a 2A self-cleavage peptide.

Mouse ES cell *in vitro* culture, differentiation, and electrotransfection

G4 (129×C57BL/6) ES cells (from Nagy Lab) were grown and maintained in previously published methods with a little modification (George et al., 2007). We cultured ES cells in Dulbecco's Modified Eagle Medium (DMEM) with high glucose (Invitrogen), supplemented with 15% ES cell-grade FBS (Gibco), 2mM GlutaMAX, 1mM sodium pyruvate, 2mM nonessential amino acids, 0.1mM 2-mercaptophenol, 1000units/ml Leukemia Inhibitory Factor (Millipore), 3μM CHIR99021 and 1μM PD0325901 (2i).

For differentiation, we use standard embryonic bodies (EB) formation method. ESCs were cultured in non-adherent conditions at a density of 1×10^5 cells/ml for 2 days on a rotator with 40 rpm. The formed EBs were cultured on plated coated with gelatin for another 5 days before

detection lineage markers with immunofluorescence staining.

Electrotransfection was performed using Nucleofector Kits for Mouse Embryonic Stem Cells (Lonza) following manufacturer's protocol. To yield *Tbx3-2A*-GFP cells, 2×10^6 cells were transfected with 3 μg CRISPR/Cas9 expression plasmid and 1 μg donor plasmid using A-030 program. If need drug selection, 2 μg/ml puromycin was added 24 hours post transfection and removed 3 days post transfection.

T7 endonuclease I assay and indel rate analysis

G4 cells were transfected with plasmid DNA as described above and harvested 2 days post transfection. Genomic DNA of 2×10^6 cells were extracted using DNeasy Blood & Tissue Kit (Qiagen) following manufacturer's protocol.

Genomic region flanking targeting site was amplified using high-fidelity DNA polymerase (TransGen Biotech), and the product was purified with QIAquick PCR Purification Kit (Qiagen) following manufacturer's protocol. A total of 400ng of the purified PCR product was mixed with 2 μl NEBuffer 2 (NEB) and ultrapure water was added to a final 20 μl volume. Heteroduplex were formed using a Thermocycler in following program: 95 ℃ for 10min, 95 ℃ to 85 ℃ ramping at –2 ℃ /s, 85 ℃ for 1min, 85 ℃ to 75 ℃ ramping at –0.3 ℃ /s, 75 ℃ for 1min, 75 ℃ to 65 ℃ ramping at –0.3 ℃ /s, 65 ℃ for 1min, 65 ℃ to 55 ℃ ramping at –0.3 ℃ /s, 55 ℃ for 1min, 55 ℃ to 45 ℃ ramping at –0.3 ℃/s, 45 ℃ for 1min,

45 ℃ to 35 ℃ ramping at –0.3 ℃/s, 35 ℃ for 1min, 35 ℃ to 25 ℃ ramping at –0.3 ℃/s, 25 ℃ for 1min. After heteroduplex formation, add 0.5ul T7 Endonuclease I (NEB) and hold at 37 ℃ for 1 hour. The digested products were analyzed by 10% polyacrylamide gel run in 1×TBE. The indel rate was calculated with ImageJ software.

FACS analysis and ESC clone derived from single cell

ES cells were harvested 3 days post transfection by TrypLE Express digestion. The rate of GFP-positive cells was measured by MoFlo XDP flow cytometer and analyzed with Summit Software. Single GFP-positive cell was sorted into each well of the prepared 96-well plate.

Immunofluorescence staining

ES cells were fixed with freshly prepared 4% paraformaldehyde/PBS for 30min at room temperature with shaking, and permeabilized with PBS containing 0.2% Triton X-100 for 30min at room temperature. Next, to avoid non-specific reaction, the ES cells were blocked with PBS containing 5% serum for 1 hour at room temperature, cells were incubated overnight at 4 ℃ with primary antibodies against Tbx3 (sc-17871, Santa Cruz), Oct4 (sc-5729, Santa Cruz), Nanog (ab80892, Abcam), Sox2 (ab97959, Abcam). Nestin (ab6142, Abcam), SMA (ab5694-100, Abcam), Gata4 (sc-1237, Santa Cruz). After washed three times in PBS, the cells were incubated with Alexa Fluor 594 donkey anti-goat IgG (A-11058, Invitrogen), Alexa Fluor 594 Goat Anti-Mouse

IgG Antibody (A-11032, Invitrogen), Alexa Fluor 594 Goat Anti-Rabbit IgG Antibody (A-11037, Invitrogen), Alexa Fluor 488 Goat Anti-Mouse IgG Antibody (A-11001, Invitrogen) or Alexa Fluor 594 Donkey Anti-Goat IgG Antibody (A-11058, Invitrogen) for 1 hour at room temperature. At last the nucleus was labeled with DAPI. Fluorescent images were captured using a Nikon A1 Confocal Laser Microscope system.

Embryonic Microinjection

To guarantee the injection of ES cells into eight-cell stage embryo before compaction, we collected the two-cell stage embryo and cultured *in vitro*. For production of two-cell stage embryos, 6-week old females were superovulated with intraperitoneal injection of pregnant mare serum gonadotrophin (PMSG) (10 IU/mouse, Ningbo Second Hormone Factory) 48 hours before mating, and human chorionic gonadotrophin (hCG) (50 IU/mouse, Ningbo Second Hormone Factory) 2 hours before mating. At the next morning, labeled the female with vaginal plugs (0.5 days post coitum, dpc). Two-cell stage embryos were flushed from oviduct of plugged females at 1.5dpc in M2 medium (Millipore), and cultured at 37℃/5% CO_2 in KSOM medium (Millipore) covered with mineral oil (Sigma). Microinjection was performed at the next day (2.5dpc). Zona pellucida (ZP) was perforated using micropipette with the help of Piezo (PMM-150FU Piezo, Sutter instrument). About fifteen ES cells were

introduced into perivitelline space through the perforation in the ZP. Injected embryos were cultured to develop into blastocyst *in vitro* as described above.

Embryo transfer

We used CD1 females mated with vasectomized CD1 males as recipients for injected embryos. We usually prepared two kinds of recipients which were 2.5dpc and 0.5dpc, depending on the development stage of injected embryos. For blastocyst stage, twelve to fifteen embryos were transferred into uterus of 2.5dpc pseudopregnan CD1 females and morula stage embryos into oviduct of 0.5dpc recipients.

Southern blot analysis

Genomic DNA of cells and tails of mice were extracted using the method of phenol-chloroform extraction. A total of 5μg genomic DNA was separated on a 0.7% agarose gel after digested by BglII (NEB), and then transferred to a nylon membrane (Roche) and hybridized with PCR based Dig(Roche)-labeled probes.

Immunohistochemistry

After fixation in 4% PFA, the embryo was dehydrated in a series of grade ethanol, embedded in paraffin and cut into section. Heat induced epitope retrieval was performed using citrate buffer (pH 6.0). For detection of GFP, we use anti-GFP rabbit monoclonal antibody (2956S, Cell Signaling) at 1:200 dilution rate.

Real-time (quantitative) PCR

Total RNA was extracted using RNeasy Mini Kit (Qiagen) in accordance with the mannufacturer's protocol. cDNA were synthesized with oligo-dT primer by M-MLV Reverse Transcriptase Kit (Promega). Q-PCR reactions were performed using the SYBR Green I Master Mix and LightCycler 480 (Roche). Gene-specific primers for Q-PCR can be found at previous study (Han et al., 2010).

Prediction and detection of potential off targets

We screened potential off target sites around the mouse genome (mm10) with CasOT software (Xiao et al., 2014) based on the role: screened potential target sites allowing for up to three base pair mismatches compared with sgRNA and the other three different PAM in the first base pair (Hsu et al., 2013). Genomic DNA regions around potential off target sites were amplified, purified and analyzed by T7EN I analysis as described above.

Statistical analyses

Student's t-tests were used to compare differences between any two groups.

Table S1. Off-target Analysis, Related to Figure 1 and S3.

Site name	Coordinate	Sequence	Indel mutation frequency (Mutant/Total)
Tbx3	5:119683061-119683083:+	AGCCAGACAGGTCTTGCAGC-GGG	/
OT1	1:172492216-172492239:+	AG**a**C**g**GACAGG**c**CTTGCAGC-**t**GG	0/10
OT2	1:189903526-189903549:+	AG**a**CAGACAGGTCT**g**GCAG**g**-GGG	0/10
OT3	3:89081084-89081107:+	AGCCAGACAGG**a**CTT**c**CAG**t**-GGG	0/10
OT4	4:140147710-140147733:+	AGCCAGA**a**AGGTCT**g**GCAG**t**-**t**GG	0/10
OT5	5:114444949-114444972:+	AG**a**CAGACAGG**c**CTTGC**t**GC-**t**GG	0/10
OT6	8:54554792-54554815:-	AGCCAGACAG**t**T**gc**TGCAGC-**a**GG	0/10
OT7	10:86070612-86070635:-	AGCCA**a**ACAGGT**a**T**g**GCAGC-**t**GG	0/10
OT8	11:84710571-84710594:+	AGCCAGA**g**AGG**g**CTTG**t**AGC-**a**GG	0/10
OT9	12:110269346-110269369:+	AGCCAGACAGG**g**CTT**c**C**t**GC-**a**GG	0/10
OT10	19:27716654-27716677:+	AGCCA**c**ACAGG**g**CTTGCA**a**C-**a**GG	0/10
OT11	X:166592140-166592163:-	AG**a**CAGACA**a**G**c**CTTGCAGC-aGG	0/10

Mismatches between potential off-target site and on-target sequence are shown in lower-case, boldface and underlined. Sequence of PAM and sgRNA is separated by hyphen. Coordinate shows the location of potential off-target site in mice genome. Indel mutation frequencies in targeted mice were calculated by T7EI assay. OT indicates off-target; /, not tested.

Table S2. Oligonucleotides used for constructing sgRNA expression vector

Gene target	Direction	Sequence (5'to 3')
Tbx3	F	CACCGAGCCAGACAGGTCTTGCAGC
	R	AAACGCTGCAAGACCTGTCTGGCTC
GFP	F	CACCGCGCGCCGAGGTGAAGTTCGA
	R	AAACTCGAACTTCACCTCGGCGCG

Table S3 Oligonucleotides used for T7EI assay and genotyping

Primer	Function	Sequence (5'to 3')
GF	Genotyping Internal probe	ATGGTGAGCAAGGGCGAG
GR		TTACTTGTACAGCTCGTCCATGCCGT
OF	Genotyping T7EI assay	TCCCCACTCTCTAACTCCCTATGT
OR		GTCTCTGGAAAGGCTTTAGTGCTC
Tbx3-ex-F	external probe	TGGAAGATACTAAGATACTGTGTGC
Tbx3-ex-R		TTGGGTGACAAGGACACTGA

Table S4 Oligonucleotides used for construction of Donor plasmid

Primer	Function	Sequence (5'to 3')
Tbx3-LA-F	Left homologous arm	ATGGCGCGCCTCAAGTGCCTCAGTATCCTG
Tbx3-LA-R		TCTCCTCCACGTCACCGCATGTTAGAAGACTTCCTCTGCCCTCC GGTGAGCCACTGCAAGACCTGTCTGGCTTGG
T2A-GFP-F	2A-eGFP	GAGGGCAGAGGAAGTCTTCTAACATGCGGTGACGTGGAGGAGA ATCCCGGCCCTATGGTGAGCAAGGGCGAG
T2A-GFP-R		GGTACCTTACTTGTACAGCTCGTCCATGCCGT
Tbx3-RA-F	Right homologous arm	ATCGATAAACAAGAAAAACAAAATCGC
Tbx3-RA-R		GGTACCACAATTCAATAAATTAAAGTT

Table S5 Oligonucleotides used for off-targeted analysis

Gene target	Direction	Sequence (5'to3')
OT1-Tbx3-Stop	F	TACCATATTGCAGCCGCTTAC
	R	CTTCTTCTCACCTCCACAGTCA
OT2-Tbx3-Stop	F	AAGTCTGGAACTCGGCGTCTG
	R	GGGTGCTGGAAACTGAACCTC
OT3-Tbx3-Stop	F	TGGTTGGTGGTCCATTGTTTG
	R	AGTTTCTGCTCCATCTTTATCC
OT4-Tbx3-Stop	F	GGAAGAGTGACAGGCATTGGT
	R	ACTCACGCCCTGCTGGGTTTA
OT5-Tbx3-Stop	F	TGGCAACAGCAGTGGTAATA
	R	GAGAAACAAAGTTCCCTAGATG
OT6-Tbx3-Stop	F	TTGTGAGTCTACTGGGCTAT
	R	GTGCATTGTAACTATGAGGC
OT7-Tbx3-Stop	F	AGTATCAGGACCCAAGCCAACC
	R	AGCAGCCACCCAGTAACACG
OT8-Tbx3-Stop	F	GATTCCAAGCACAGCCCTAA
	R	TGTGAAACTGGGTCCTCCTG
OT9-Tbx3-Stop	F	GGCTTTGATAGAGGCAACTGG
	R	CTTGGGACTCGGGTCTTATGT
OT10-Tbx3-Stop	F	TGCTGGAGACAGACCACAAC
	R	CCAAACAGGGTGGCTACTTC
OT11-Tbx3-Stop	F	GCCTTTGTCTTGAGGGATGT
	R	AGTGTCCAGCACAGGATTAG

Reference

Cong, L., Ran, F.A., Cox, D., Lin, S., Barretto, R., Habib, N., Hsu, P.D., Wu, X., Jiang, W., Marraffini, L.A., *et al.* (2013). Multiplex genome engineering using CRISPR/Cas systems. Science 339, 819-823.

George, S.H., Gertsenstein, M., Vintersten, K., Korets-Smith, E., Murphy, J., Stevens, M.E., Haigh, J.J., and Nagy, A. (2007). Developmental and adult phenotyping directly from mutant embryonic stem cells. Proc Natl Acad Sci U S A 104, 4455-4460.

Han, J., Yuan, P., Yang, H., Zhang, J., Soh, B.S., Li, P., Lim, S.L., Cao, S., Tay, J., Orlov, Y.L., *et al.* (2010). Tbx3 improves the germ-line competency of induced pluripotent stem cells. Nature 463, 1096-1100.

Hsu, P.D., Scott, D.A., Weinstein, J.A., Ran, F.A., Konermann, S., Agarwala, V., Li, Y., Fine, E.J., Wu, X., Shalem, O., *et al.* (2013). DNA targeting specificity of RNA-guided Cas9 nucleases. Nat Biotechnol 31, 827-832.

Xiao, A., Cheng, Z., Kong, L., Zhu, Z., Lin, S., Gao, G., and Zhang, B. (2014). CasOT: a genome-wide Cas9/gRNA off-target searching tool. Bioinformatics.

Supplemental figure legends

Figure S1

Generation of Tbx3-2A-GFP ESCs by using the CRISPR/Cas9 system. (A) The homogeneous expression pattern of Tbx3 in the presence of 2i. Scale bars, 20 μm. (B) Flow cytometry analysis and sorting of the GFP-positive cells after electrotransfection. (C) Immunofluorescence

staining of Tbx3-2A-GFP ESCs. Cells were stained with the pluripotency markers Oct4, Sox2 and Nanog. Scale bars, 20 μm. (D) PCR screening of Tbx3-2A-GFP knock-in ESCs. OF, outside forward primer; OR, outside reverse primer; GF, internal forward primer; GR, internal reverse primer; M, molecular marker; WT, wild type. € Immunofluorescence staining of embryoid body (EB) formed from modified ESCs on day 7. Cells were stained for three germ layer markers Nestin (ectoderm), α-SMA (mesoderm) and Gata4 (endoderm). Scale bars, 20 μm.

Figure S2

Near 100% ESC-derived mice yielded by eight-cell stage embryo injection. (A) Blastocyst development from eight-cell stage embryo injected with Tbx3-2A-GFP ESCs. Scar bars, 100 μm. (B) F0 generation of reporter knock-in mice were identified by southern blot analysis. T, targeted knock-in mice. (C) F1 offsprings of Tbx3-2A-GFP mice mating with wild-type CD-1 females. (D) Summary of F1 generation of Tbx3-2A-GFP mice mating with wild-type CD-1 females. (E) GFP fluoresecen images of early embryos from F1 offsprings of reporter knock-in mice (related to Fig. S2D). Scar bars, 1mm.

Figure S3

Schematic overview of generating Tbx3-2A-GFP mice by CRISPR/Cas9 system and eight cell-stage embryo injection technology. Segments on the right side indicate time needed for each step; FACS,

Fluorescence-activated cell sorting.
